# Psychology and Mental Diseases

**Published:** 1914-10

**Authors:** 


Psychology and Mental Diseases. C. B. Burr, M. D., Medical Director of the Oak Grove Hospital for Mental and Nervous Diseases. F. A. Davis Company, Publisher; price, $1.50.

Right now when psychotherapeutics and psychic states have entered so prominently into the treatment of our patients there is a great need felt for a short but comprehensive outline of the principles of psychology and the psychoses of every-day life. Many successful busy practitioners have never had the opportunity to specially inform themselves along these lines, and yet greatly appreciate the daily need of such information. To them this little hand volume of 226 pages can be highly recommended, confidently feeling that it will not prove a disappointment.

The chapters on psychology are clear, brief and quite ample to illustrate normal and mental processes. Such knowledge is essential in understanding the moods and temperaments of patients and especially in comprehending the pathological states of mind.

The differentiation and treatment of the psychoses is modern, precise, and eminently practical to guide one in the diagnosis and management of such cases.

An ingenious chapter on Symbolism in Sanity and in Insanity is added which is both entertaining and instructive. Freud's researches have been recognized in part and due consideration is given to heredity and syphilis in the etiology of mental diseases.

M. D.

We have received for review a copy of Volume IV of the new, third edition of the "Reference Handbook," just published by Wm. Wood & Co. of New York. The fourth volume, just issued, is in no way inferior to its predecessors. Of the 444 individual articles contained in Volume IV are conspicuous for their especial excellence those on: Surgery of the Erophagus; Evolution; Eye; Anatomy, Dioptrics, Injuries, Tumors; Eyestrain, Excisions, Fietus, Development and Diseases; Club-foot and other Distortions and Disabilities of the Foot; Fractures; Fungi, Food and Drug Laws; Medical Ethics; Gunshot Wounds. The short definitions of terms more exhaustively treated in other articles, is a feature of great usefulness to the busy practitioner.

